# Global burden of osteoarthritis in adults aged 30 to 44 years, 1990 to 2019: results from the Global Burden of Disease Study 2019

**DOI:** 10.1186/s12891-024-07442-w

**Published:** 2024-04-19

**Authors:** Yixiang He, Wenkai Jiang, Wenji Wang

**Affiliations:** 1 The First Clinical Medical College of Lanzhou University, Lanzhou, 730000 Gansu China; 2 The Second Clinical Medical College of Lanzhou University, Lanzhou, 730000 Gansu China; 3 Department of orthopedics, the First Hospital of Lanzhou university, Lanzhou, 730000 Gansu China

**Keywords:** Osteoarthritis, Global Burden of Disease, Years lived with disability, Incidence, Prevalence, Young adult

## Abstract

**Background:**

Osteoarthritis (OA) is a common orthopedic disorder, and its incidence has been increasing among young adults in recent years. The purpose of this study is to investigate the global, regional, and national trends in OA burden and variation among individuals aged 30 to 44 from 1990 to 2019.

**Methods:**

Data on the incidence, prevalence, and years lived with disability (YLDs) related to OA were sourced from the Global Burden of Disease Study 2019 among individuals aged 30 to 44. These measures were stratified by gender, region, country, and socio-demographic index (SDI). Additionally, we analyzed YLDs attributable to risk factors.

**Results:**

In 2019, there were a total of 32,971,701 cases of OA among individuals aged 30 to 44 years worldwide, with an additional 7,794,008 new incident cases reported. OA of the knee was the primary contributor to both incidence and prevalence rates over the past three decades. From 1990 to 2019, both males and females in countries with high SDI and high-middle SDI showed upward trends in age-standardized incidence, prevalence, and YLDs rates. In 2019, the United States of America had the highest age-standardized incidence, prevalence, and YLDs rates. Elevated body-mass index (BMI) was found to be the most prevalent risk factor for osteoarthritis-related YLDs. Age-standardized YLDs rates were positively associated with SDI.

**Conclusions:**

OA remains a significant disease burden on individuals aged 30 to 44, with modifiable risk factors such as unhealthy lifestyle and obesity representing key targets for future interventions aimed at reducing the impact of this condition on younger generations.

**Supplementary Information:**

The online version contains supplementary material available at 10.1186/s12891-024-07442-w.

## Introduction

Osteoarthritis (OA) is a prevalent chronic joint disease resulting from an imbalance in the intra-articular environment caused by various factors, leading to the degeneration of joint cartilage and other associated tissues, accompanied by the activation of pro-inflammatory responses [1, 2]. OA can affect any joint in the body, although it is most commonly observed in the knee, hip, interphalangeal joints, and spine. Patients typically experience symptoms such as joint pain and dysfunction, which may even lead to disability. Recurrent discomfort can also significantly impact patients’ mental health, leading to anxiety, depression, and opioid misuse, which substantially compromises their overall quality of life [3–5].

With the global aging phenomenon and increasing obesity rates, there is a gradual escalation in the disease burden of OA within the world population [1]. Previous research on OA has predominantly focused on the elderly population, with findings indicating significant positive associations between age and the incidence of knee and hip OA [6]. In contrast, the population aged 30–44, which constitutes the predominant labor force in society, possesses a robust demographic foundation and demonstrates an increased vulnerability to pathogenic factors. In terms of treatment, joint replacement is a commonly utilized method for treating end-stage OA, younger patients demonstrate a higher propensity to require revision surgery and experience an increased incidence of postoperative complications, thus resulting in a greater disease burden [7, 8]. However, the limited research conducted on this specific population has resulted in a scarcity of knowledge regarding the risk factors and optimal management strategies for younger patients. In recent decades, there has been an observable trend of OA affecting a younger population. A retrospective study of 290,897 OA patients revealed a significant rise in the proportion of younger individuals aged below 44 years, from 2001 to 2018 [5]. The precise role of age in the pathogenesis of joint disease remains unclear and may be attributed to other risk factors, such as obesity and joint damage, whose effects accumulate over time. Previous studies have investigated the global prevalence trend of knee, hip, and hand OA in relation to high BMI; however, there is a dearth of systematic discussion on the trends of OA in young adults, its global burden of disease, and related risk factors [9–11]. Therefore, conducting further comprehensive research is imperative to gain a profound understanding and provide an accurate depiction of the prevalence trends and risk factors associated with OA among young individuals.

The Global Burden of Disease study (GBD) provides comprehensive data on hundreds of diseases across numerous countries and regions, enabling a thorough evaluation of long-term trends in disease prevalence based on epidemiological characteristics such as geography, age, gender, and socio-demographic index (SDI). It also quantifies the health loss and disease burden caused by these illnesses. Therefore, in this study, we conducted an analysis of the incidence, prevalence and years lived with disability (YLDs) associated with OA among patients aged 30–44 years utilizing data extracted from the GBD database spanning the period between 1990 and 2019. In this process, the health burden and epidemiological trends of young individuals with OA were evaluated, while also describing differences in disease distribution based on levels of human development and geographical location. By stratifying global trends by gender and SDI, the primary risk factors, specifically obesity, were identified. This study aims to comprehensively investigate the incidence trend of OA within this age cohort, thereby providing valuable insights for designing disease prevention strategies tailored specifically for this population.

## Materials and methods

### Resources

The data for this study are accessible via a publicly available open-access repository. The datasets utilized for the analyses can be obtained through the GBD Data Input Sources Tool (https://ghdx.healthdata.org/gbd-2019/data-input-sources) and GBD Result Tool (https://vizhub.healthdata.org/gbd-results/).

We downloaded data from the GBD 2019 by sex, year, age and location, including incidence, prevalence, and YLDs. GBD 2019 provide 369 diseases and injuries, and 87 related risk factors [12]. In this study, we identified osteoarthritis with the following International Classification of Diseases and Injuries codes: M16-M16.9 for osteoarthritis hip, M17-M17.9 for osteoarthritis knee and M18-M18.9 for osteoarthritis hand. For age groups, we selected people aged 30 to 44 years with five-year age intervals (30 to 34, 35 to 39 and 40 to 44).

### Modeling

The data analysis and selection processing have been mentioned in previous a publication [13]. After utilizing standardized tools to estimate the incidence of cases across various age groups, genders, locations, and years, the processed data can be subjected to modeling for a wide range of diseases and injuries. The DisMod-MR tool was utilized in this study to model GBD data, enabling a comprehensive evaluation of all available information on disease incidence, prevalence, remission, and mortality rates while promoting coherence among epidemiological parameters.

### Socio-demographic index (SDI)

SDI, ranging from 0 to 1, is an index to assess the level of social development in a region or country [14]. GBD 2019 divided all countries/territories into five levels: high SDI, high-middle SDI, middle SDI, low-middle SDI and low SDI. To further explore the relationships between SDI and disease burden, we analyzed the association of age-standardized YLDs rates and SDI values in 2019 across 21 GBD regions.

### Attributable risk

The GBD 2019 classification categorizes the included risk factors into three primary domains: (1) environmental and occupational, (2) behavioral, and (3) metabolic. The GBD comparative risk assessment framework, proposed by the GBD Risk Factor Collaborators, was utilized in this study to evaluate the proportion of deaths and disability-adjusted life years (DALYs) attributed to various risk factors. This comprehensive framework encompasses six major steps and is employed for calculating disease burden specific to each individual risk factor [12]. For osteoarthritis in GBD 2019, high body-mass index (BMI) was the most important risk factor (high BMI was defined as BMI > 25 kg/m^2^).

### Disability-adjusted life years (DALYs)

DALYs are a standardized metric utilized to quantify the burden of disease. Given that the cause-of-death model in GBD estimation does not assume a causal relationship between OA and mortality, DALYs for OA are considered equivalent to years lived with disability (YLDs). YLDs can be calculated by multiplying the prevalence of each severity level with their respective disability weights.

### Statistical analysis

Statistical analysis was performed by using R software (Version 4.2.2). The age-standardized rate (ASRs) of incidence, prevalence, YLDs per 100,000 people, and their corresponding 95% confidence intervals (CIs) were calculated from 1990 to 2019 in GBD 2019 using the world standard population obtained from the GBD 2019 analytical tool website [14]. Temporal trends of OA burdens were estimated using the Joinpoint software (Version 4.9.1.0) regression model to calculate annual percentage change (APC) and average annual percentage change (AAPC) [15]. The AAPC values can indicate both the magnitude and direction of temporal trends. If AAPC > 0, it suggests an increasing trend in the ASRs during this study period; otherwise, it indicates a decreasing trend. All rates are reported per 100,000 populations. The “ggplot2” package in R was used for visualization. *P* values < 0.05 were considered to be statistically significant.

## Results

### Diseased joints

From 1990 to 2019, there was an upward trend in the incidence and prevalence of OA among individuals aged 30–44 years of both sexes, with a significantly higher incidence and prevalence observed among female patients than among male patients.

**Fig. 1:**
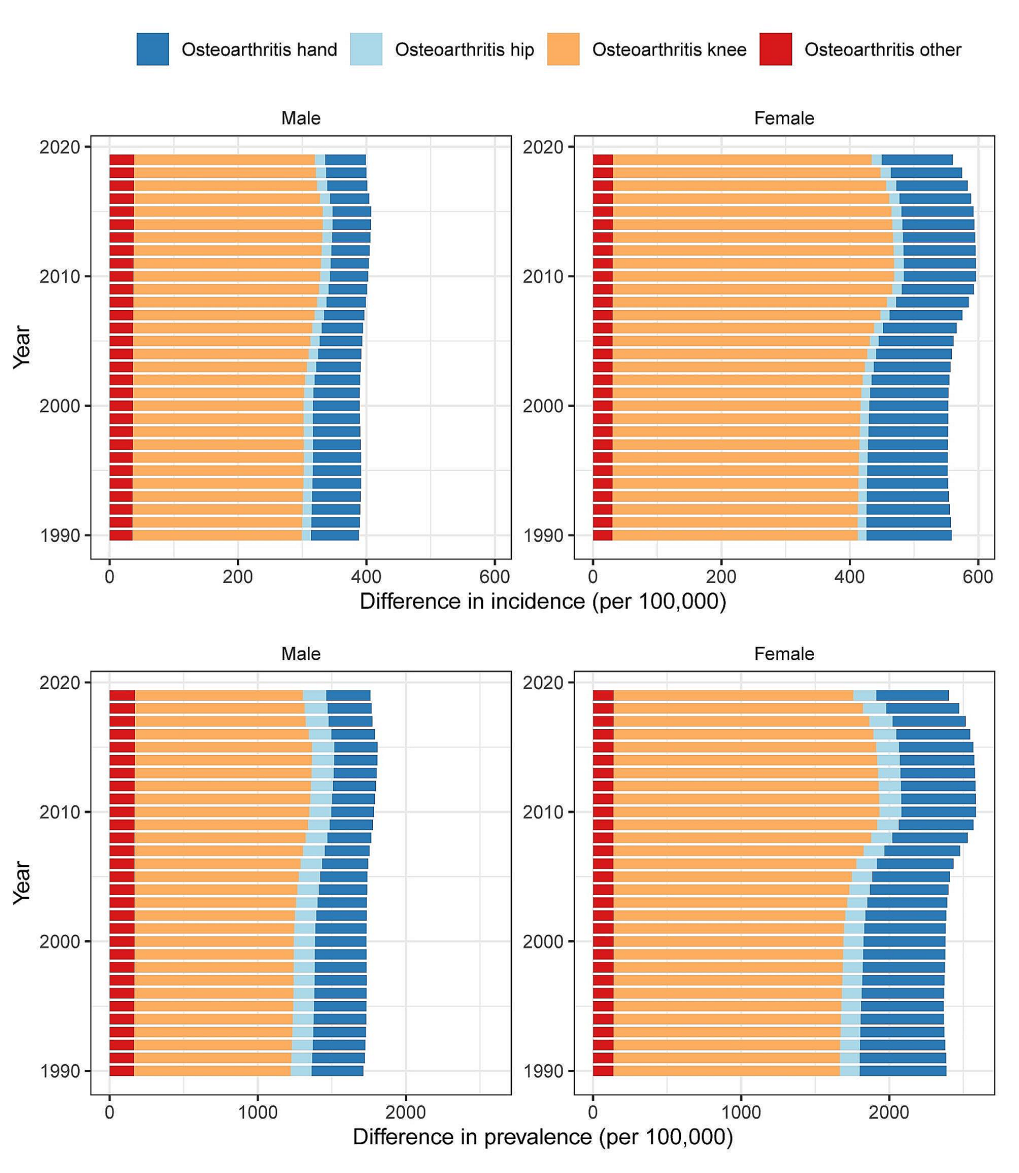
Trends in the incidence and prevalence of osteoarthritis across different joint sites among aged 30–44, stratified by gender from 1990 to 2019

Additionally, knee OA was identified as the primary joint affected by disease, with a significantly higher incidence and prevalence rate compared to other joints, followed by hand OA. (Fig. 1)

### Global trends

Globally, in 2019, the estimated number of OA cases among individuals aged 30–44 years was 32,971,701 [95% UI: 26,722,308 to 39,931,878], which represents a notable increase of 7,794,008 [95% UI: 6,297,762 to 9,584,972] new cases compared to 1990.

The incidence of OA among individuals aged 30–44 years increased from 471.86 in 1990 to 478.97 in 2019, with an average annual percentage change (AAPC) of 0.05 [95% CI: 0.03 to 0.07], and the growth trend exhibits a notable discrepancy (*P* < 0.001). Meanwhile, the prevalence of OA in this population increased from 1989.90 in 1990 to 2027.07 in 2019, with an AAPC of 0.07 [95% CI: 0.03 to 0.12], *P* < 0.05. The YLDs also increased from 67.29 in 1990 to 68.62 in 2019, with an AAPC of 0.08 [95% CI: 0.03 to 0.12], *P* < 0.05.

When grouped by gender, the AAPC of OA incidence was 0.08 [95% CI: 0.06 to 0.11] in males and 0.02 [95% CI: -0.04 to 0.08] in females. The prevalence of OA in males demonstrated an AAPC of 0.09 [95% CI: 0.06 to 0.12] and 0.03 [95% CI: -0.01 to 0.07] in females. The incidence, prevalence, and YLDs rate of male patients all showed an increasing trend, while those of female patients increased slightly but without statistical significance.

From 1990 to 2019, the age-standardized incidence, prevalence, and YLDs rates of young individuals in various SDI regions increased to varying degrees in different SDI groups. Among them, the most notable increase is observed in low SDI and low-middle SDI areas, with a growth rate exceeding 100%. (Table 1; Table S1-S3)

After further stratification of individuals aged 30–44 years by age group, it is evident that the age-standardized incidence, prevalence and YLDs rates for all subgroups (30–34; 35–39; 40–44) have consistently increased globally over the past three decades from 1990 to 2019.

**Table 1:** Change trend of the global burden of osteoarthritis, 1990 to 2019

	Incidence (95%CI)	*P*	Prevalence (95%CI)	*P*	YLDs (95%CI)	*P*
Global						
AAPC of ASR	0.05(0.03 to 0.07)	< 0.001	0.07(0.03 to 0.12)	0.001	0.08(0.03 to 0.12)	0.001
Percentage change of number	66.51%		69.31%		69.40%	
High SDI						
AAPC of ASR	0.09(0.07 to 0.11)	< 0.001	0.18(0.15 to 0.21)	< 0.001	0.18(0.15 to 0.21)	< 0.001
Percentage change of number	18.52%		22.00%		21.96%	
High-middle SDI						
AAPC of ASR	0.1(0.04 to 0.15)	< 0.001	0.08(0.03 to 0.13)	0.003	0.09(0.04 to 0.14)	0.001
Percentage change of number	48.08%		50.39%		50.62%	
Middle SDI						
AAPC of ASR	0.14(0.1 to 0.17)	< 0.001	0.16(0.13 to 0.19)	< 0.001	0.16(0.13 to 0.19)	< 0.001
Percentage change of number	80.57%		85.57%		85.41%	
Low-middle SDI						
AAPC of ASR	0.25(0.21 to 0.28)	< 0.001	0.26(0.23 to 0.3)	< 0.001	0.28(0.23 to 0.32)	< 0.001
Percentage change of number	109.07%		112.60%		113.20%	
Low SDI						
AAPC of ASR	0.18(0.16 to 0.2)	< 0.001	0.19(0.17 to 0.21)	< 0.001	0.2(0.18 to 0.22)	< 0.001
Percentage change of number	150.56%		152.52%		153.50%	

Abbreviations: ASR, age-standardized rate; AAPC, average annual percentage change; YLDs, years lived with disability; SDI, socio-demographic index; CI, confidence interval

### Regional trends

Further analysis of the 21 countries and regions included in the Global Burden of Disease Study in 2019 reveals that young adults aged 30–44 years in high-income North America, Australasia, and high-income Asia Pacific regions have the highest age-standardized incidence rates of OA, with rates of 792.84, 649.49, and 626.58, respectively. In contrast, the age-standardized incidence rates were found to be lowest in Central Asia (384.99), Central Europe (356.90), and Southeast Asia (332.62). (Table S1)

**Fig. 2:**
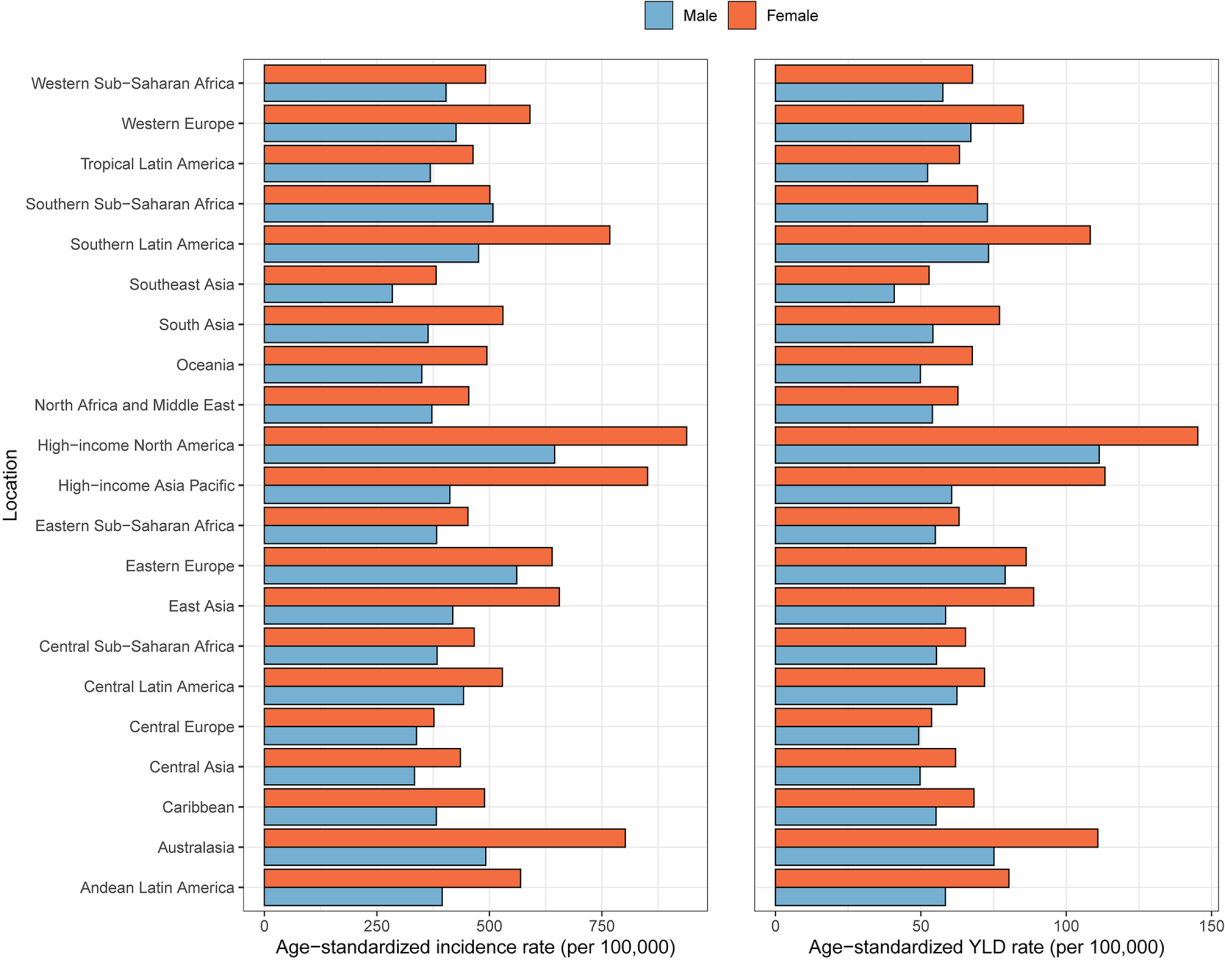
Age-standardized incidence and YLDs rates of osteoarthritis in young adults in different regions

Meanwhile, the regions with the highest age-standardized YLDs rate were high-income North America (128.44), Australasia (93.30), and southern Latin America (91.10), while Southeast Asia (46.78), Central Europe (51.43), and Central Asia (55.90) had the lowest age-standardized YLDs rates. Similar patterns in distribution were observed when stratified by sex, with female patients exhibiting significantly higher age-standardized incidence and YLDs rates than their male counterparts. (Table S3; Fig. 2)

### National trends

In 2019, the United States of America had the highest age-standardized incidence, prevalence and YLDs rates of OA in young adults aged 30–44 years worldwide, with rates of 816.41, 3933.78 and 132.42, respectively. Following that, Iceland (702.89; 3256.53; 110.81) and the Republic of Korea (734.73; 2988.98; 102.48) come in second and third place, respectively.

**Fig. 3:**
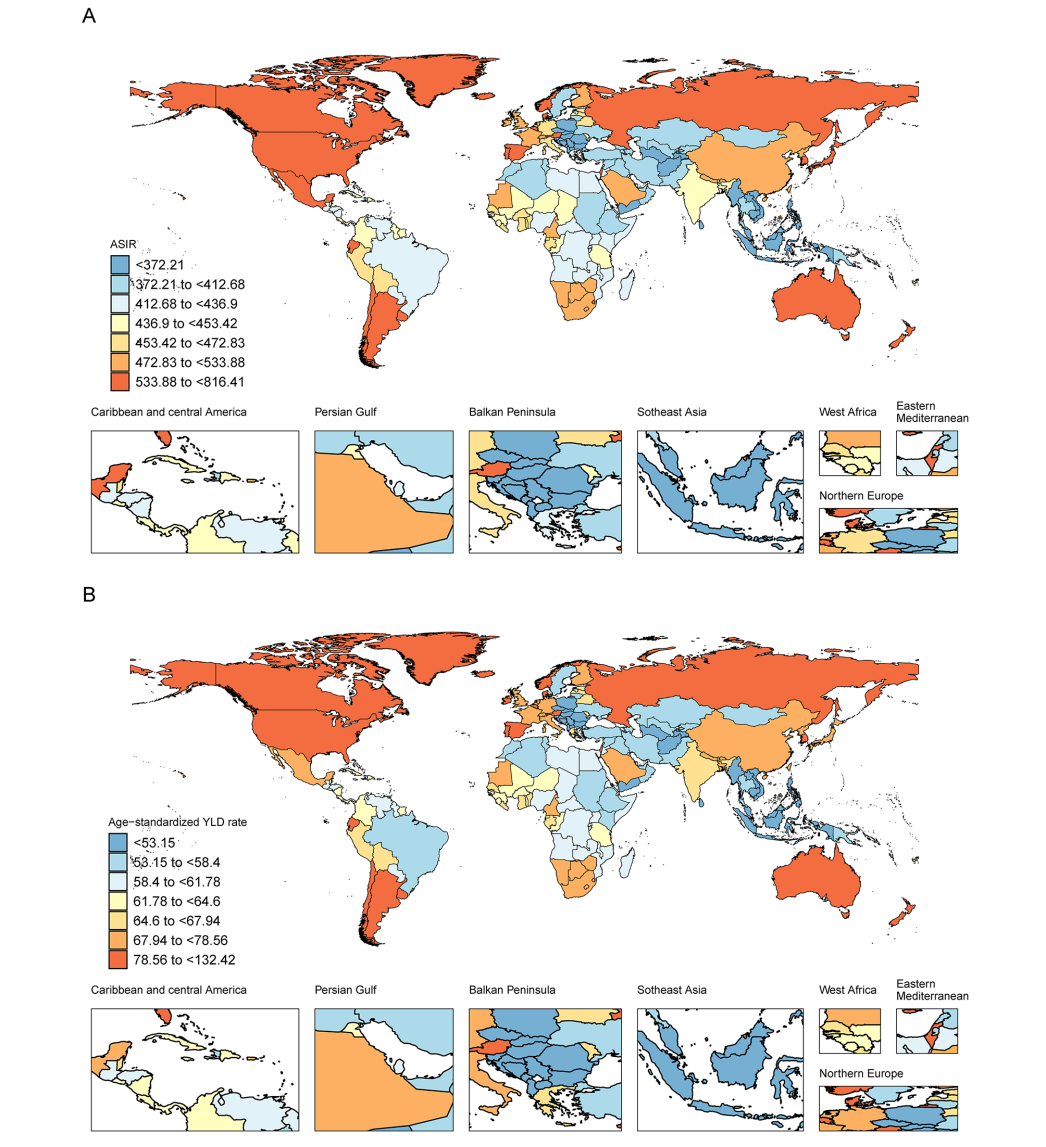
Age-standardized incidence of osteoarthritis (**A**) and age-standardized YLDs rates (**B**) in young adults worldwide in 2019, by country

On the other hand, the Philippines (297.72; 1227.81; 41.80), Indonesia (322.27; 1319.57; 44.97), and Timor-Leste (321.88; 1331.29; 45.46) are situated at a lower level. (Table S4; Fig. 3)

### Global trends by SDI

**Fig. 4:**
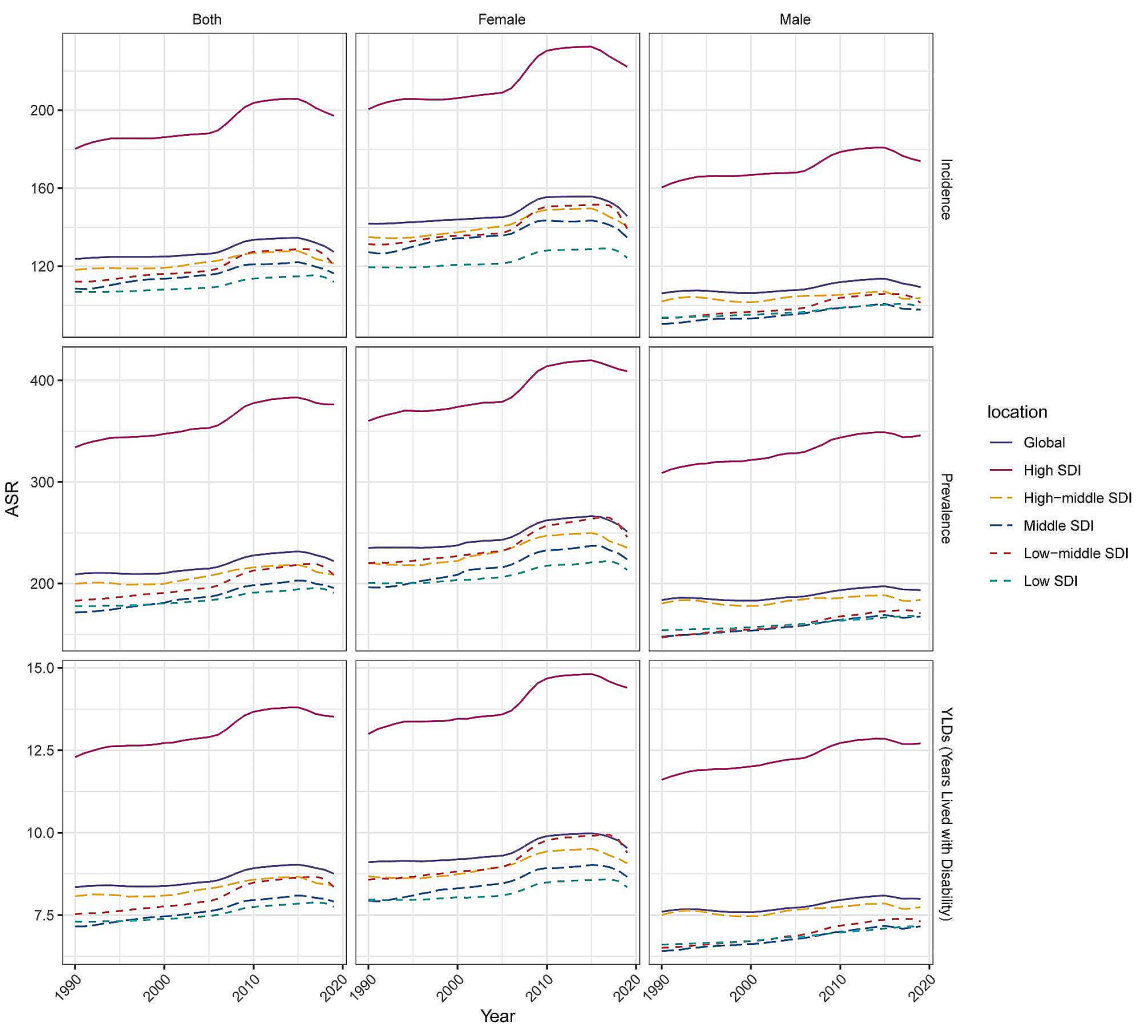
Gender and SDI-specific trends in age-standardized incidence, prevalence, and YLDs of osteoarthritis among young individuals

After considering different SDI factors, the incidence, prevalence and YLDs rates of OA among individuals aged 30–44 years have significantly increased in regions with varying levels of SDI over the past three decades. Among them, regions with high SDI exhibited significantly higher values in each index compared to regions with low SDI, and this trend gradually increased over time. Similar patterns were also observed in other SDI groups. (Fig. 4; Table S1-S3)

**Fig. 5:**
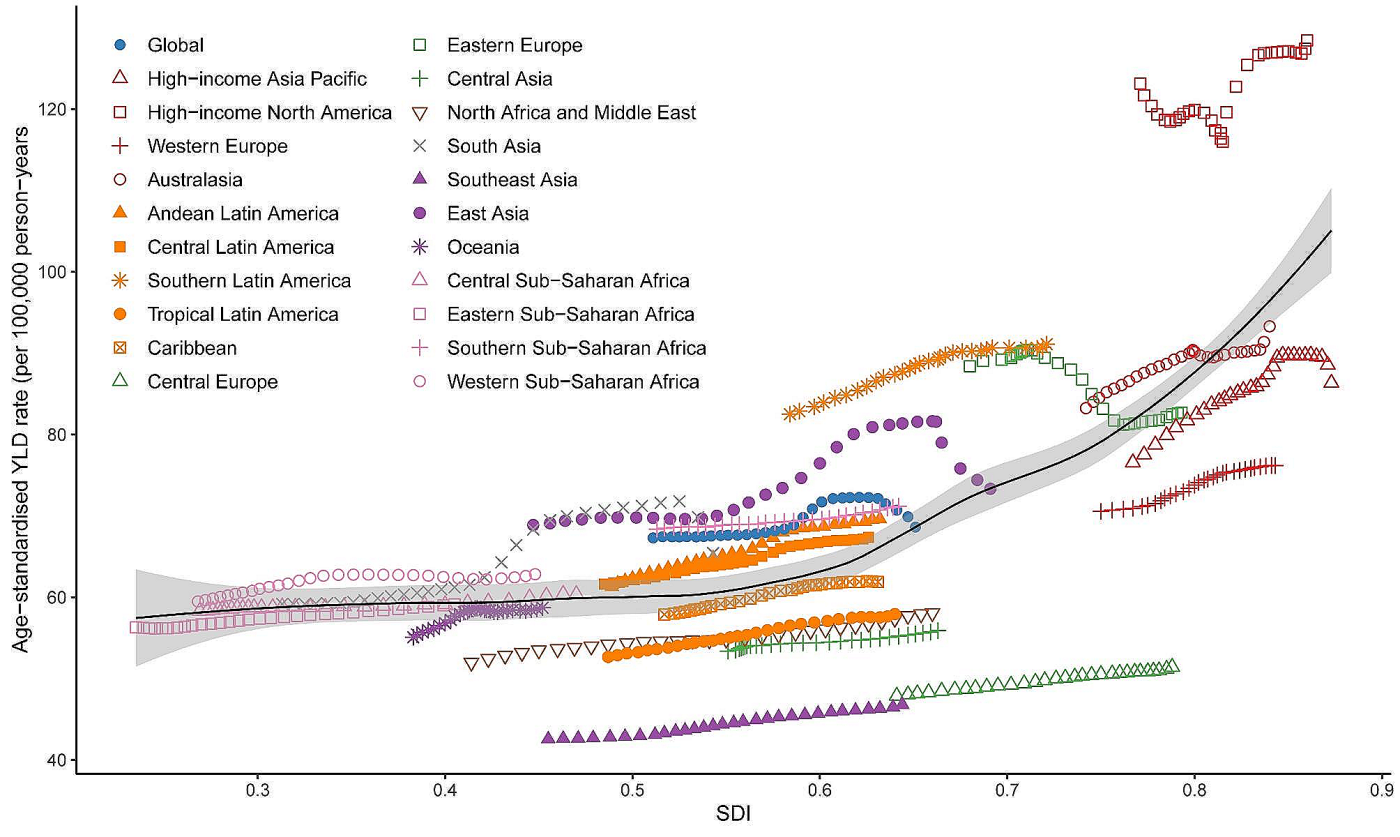
The trend in age-standardized YLDs and socio-demographic index associated with osteoarthritis in young people in 21 global burden of disease (GBD) regions, 1990–2019

In 2019, there was a positive associations between age-standardized incidence, prevalence, and YLDs rates and SDI in most GBD countries and regions, increasing with SDI. Globally, the index has shown a consistent upward trend from 1990 to 2019, with a temporary decline approximately 2015 and subsequent gradual convergence toward the expected level in terms of SDI. Regions with high SDI levels, such as high-income North America, southern Latin America, Australasia, and high-income Asia Pacific, have long-term age-standardized YLDs that are higher than the world average. Conversely, North Africa and Middle East, Southeast Asia and Central Latin America demonstrate slightly lower YLDs rates. (Fig. 5)

### Risk factors

In individuals aged 30–44 years, high BMI remains a significant risk factor for OA and exerts a substantial impact on the burden of disease measured by YLDs caused by OA.

**Fig. 6:**
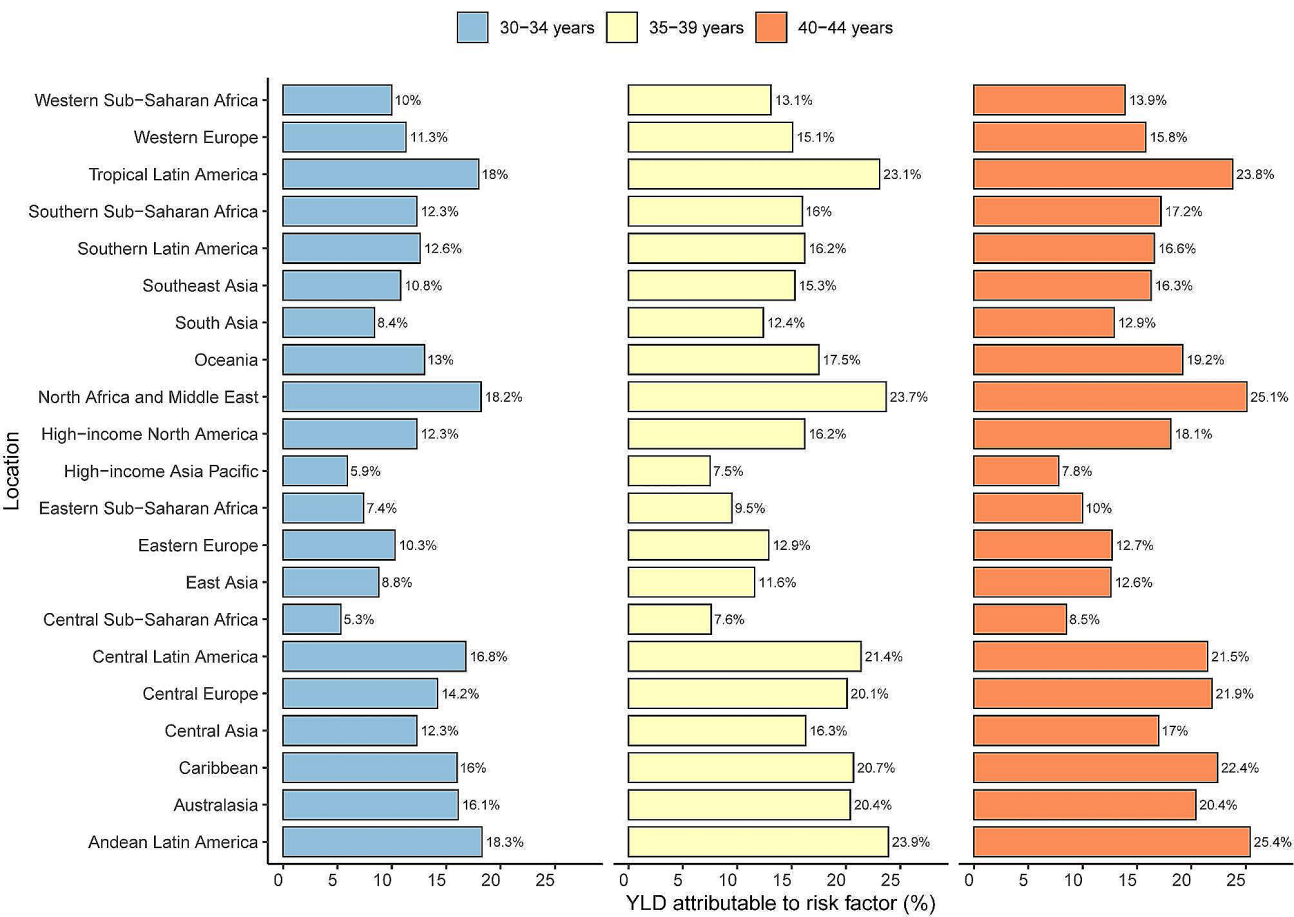
Percentage of YLDs caused by osteoarthritis attributed to high BMI in Global Burden of Disease study in 2019, by age group

In 2019, the age-standardized rates of YLDs for OA caused by high BMI gradually increased with patient age in most regions and different age subgroups, reaching the highest levels in the 40–44 age group. Regionally, the regional attributable risk of high BMI ranged from 5.3% to 25.4% across all age groups. Andean Latin America exhibited the highest YLDs attribution rates among each age groups, with percentages of 18.3%, 23.9%, and 25.4% respectively, followed by North Africa and Middle East (18.2%, 23.7%, 25.1%) and Tropical Latin America (18%, 23.1%, 23.8%). The regions with the lowest attributions of high BMI to OA in young people were Central Sub-Saharan Africa (5.3%, 7.6%, 8.5%) and High-income Asia Pacific (5.9%, 7.5%, 7.8%). (Fig. 6)

## Discussion

This study aims to describe the burden of OA and its changing trends among individuals aged 30–44 years globally, regionally, and nationally by sex and SDI between 1990 and 2019. Over the past three decades, there has been a significant increase in both the incidence and prevalence rates of OA within this age group compared to 1990. The age-standardized rates demonstrated an upward trajectory across various SDI regions, with significantly higher incidence, prevalence, and YLDs rates observed in high SDI regions compared to other regions. Furthermore, a high BMI remains a significant risk factor for OA and has a substantial impact on the disease burden as measured by YLDs attributed to OA.

While the elderly population remains the primary focus of OA research, it is imperative to pay greater attention to the escalating prevalence among younger individuals. According to the definition of World Health Organization (WHO), individuals aged below 44 are classified as young adults, representing the primary workforce in society and thus constituting the central focus of attention in this study. Graham [5] investigated the demographic characteristics of OA patients from 2001 to 2018 and observed a gradual increase in the proportion of younger patients aged between 18 and 44, rising from 6.2% in 2001 to 22.7% in 2018, while most patients were still elderly. Our study cohort was restricted to individuals within the age range of 30–44 years, presenting a narrower scope in comparison with Graham’s sample. Nevertheless, the obtained research outcomes exhibit a similar trend, indicating an increasing impact of OA on younger individuals.

Although there is insufficient evidence to explain why OA is on the rise in younger people, some potential causes have been hypothesized. First, As the predominant demographic in the workforce, the age cohort experiences increased stress on their joints due to daily activities, making them more susceptible to symptoms associated with OA. With the progression of economic status and a shift in people’s attitudes toward healthcare, individuals are paying more attention to their health status and actively seeking medical examination and treatment even for minor discomforts, thereby facilitating early detection and diagnosis of joint lesions. Meanwhile, with the advancement of medical standards, the likelihood of delayed diagnosis and treatment of diseases is reduced, making it easier to detect and diagnose diseases at an early stage [5]. This may partly account for the incidence of OA in younger populations being higher in high SDI regions such as high-income North America and high-income Asia Pacific than in other regions. In these regions are equipped with a relatively comprehensive medical security system that facilitates patients’ access to diagnosis and treatment. Consequently, in comparison to less developed areas, these regions can provide more reliable and efficient data [16].

Both the female gender and obesity are recognized as significant contributors to the risk factors of OA. The association between obesity-related factors and knee OA has been largely established since the 1980s [17]. The risk of knee OA in overweight individuals is 2.5–4.6 times higher than that of the general population, with a 35% increased risk for every 5 kg/m2 increase in BMI [18]. Zhang [19] confirmed a positive association between BMI and OA, particularly in the knee joint. Reyes [20] revealed that overweight and obesity significantly increase the risk of developing OA. Moreover, individuals with a BMI ranging from 25 kg/m2 to 30 kg/m2 (overweight), 30 kg/m2 to 35 kg/m2 (obesity class I), and over 35 kg/m2 (obesity class II) exhibit approximately 2, 3.1, and 4.7- times higher risks of developing knee OA than those with normal weight. Salis [21] also found that a patient’s risk of undergoing knee replacement was positively associated with their BMI. Although the associations between high BMI and OA have been confirmed by studies, the exact mechanism of action remains unclear. It is generally believed that weight gain leads to an increase in load on bearing joints, resulting in cartilage damage, which is also a key factor contributing to the high incidence of knee OA [22]. Additionally, it is believed that the inflammatory response and metabolic abnormalities resulting from obesity contribute to OA development [23].

Over recent decades, the prevalence of obesity has been increasing among individuals of all ages, including children and young adults [24]. It increases the susceptibility to chronic ailments such as OA in younger populations [25, 26]. In this study, it was found that high BMI remains the predominant risk factor for YLDs in young OA patients aged 30–44 years. Geographically, high BMI contributes to more than 20% of YLDs in Latin America, Australasia, North Africa and the Middle East. The upward trend in obesity rates in these regions has been confirmed, as demonstrated by Australia where the overall prevalence of obesity among the population increased from 19.1% in 1995 to 27.2% in 2012 [27]. Among developing countries in Latin America, the obesity rates among adolescents aged 5–19 are 41.8% in Mexico, 22.1% in Brazil, and 19.3% in Argentina [25]. A similar result was demonstrated in an analysis of a globally pooled study encompassing 128.9 million individuals [28].

The study revealed another important finding, which indicates a slight increase in the incidence rate among female patients, while both the incidence and prevalence rates among male patients exhibit a upward trend with statistical significance. These findings suggest a gradual escalation in the proportion of male patients with OA. Additionally, Graham [5] also observed a significant upward trend in the proportion of male patients over the past three decades, with an increase from 34.6% in 2001 to 39.2% in 2018, while the percentage of female patients decreased from 65.3% to 60.8%. The result can be attributed to multiple factors, including the significant increase in BMI among male individuals. The results of a recent study have revealed that the global prevalence of obesity among females has increased from 8.8% in 1990 to 18.5% in 2022, while the corresponding rates for males have risen from 4.8% to 14.0%, indicating a more significant upward trend [29]. In China, a population survey of 645,223 participants also revealed that the rate of BMI increase among males was significantly higher than that among females from 2004 to 2018 [30]. This factor has the potential to significantly contribute to the increasing prevalence of male patients with osteoarthritis, exhibiting notable differences.

Unhealthy dietary patterns and a sedentary lifestyle are significant factors in the escalating prevalence of obesity among children and adolescents [31–33]. Ultra-processed foods, with delicious taste, convenience and availability, have become increasingly prevalent among children and young people. However, they often contain high levels of salt, fat and sugar or are low in dietary fiber, resulting in imbalanced nutrition that may contribute to weight gain among consumers [34–36]. Long-term consumption of ultra-processed foods is associated with an increase in energy intake, estimated at approximately 500 kcal per day, and an average weight gain of 0.9 ± 0.3 kg after two weeks [37]. Machado [38] found a significant dose–response associations between the consumption of ultra-processed foods and obesity rates. The highest quintile of intake exhibited significantly higher BMI, waist circumference, and obesity rates than the lowest quintile. This trend was observed across all age groups, sexes, and physical activity levels.

Simultaneously, during sedentary activities such as watching television, individuals may find it more convenient to consume these food items, resulting in an excess of energy intake, particularly in regions with higher economic development, where there is greater accessibility to ultra-processed foods [34, 39, 40]. This could potentially explain why countries and regions with a higher SDI tend to experience a greater burden of osteoarthritis-related diseases.

The implementation of health management strategies specifically targeting individuals with a high BMI is a crucial intervention applicable to patients with OA, resulting in favorable outcomes. Bartholdy [41] conducted an 8-week intensive dietary intervention (IDI) that targeted patients with knee OA accompanied by overweight or obesity. A full meal-replacement diet was implemented to effectively facilitate weight reduction, resulting in significant improvements in OA symptoms among the participants. Simultaneously, active weight management can also reduce perioperative complications in obese patients [42].

Hence, it is crucial to implement measures aimed at alleviating the increasing prevalence of obesity. For example, at the national level, policies can be implemented to mitigate the rising prevalence of obesity, particularly among children and young people. In May 2010, the WHO passed resolution WHA63.14 aimed at restricting the promotion of unhealthy foods and non-alcoholic beverages to children and adolescents [43]. In American countries, governments have implemented various regulations to restrict dietary patterns while promoting the development of sports facilities and increasing physical activity levels [44, 45]. However, there are still some deficiencies in the implementation of the current policy, and sustained efforts will be required in the future to achieve the objective of reducing obesity rates and enhancing population health.

This study provides a systematic description of the disease burden of OA in individuals aged 30–44 years, stratified by gender and SDI region. Additionally, it analyzes diets, policies, and lifestyles across regions with varying disease burdens to offer scientific guidance for countries developing food and healthcare policies. However, it still has certain inherent limitations. First, in terms of analyzing risk factors, the GBD database offers limited data on such factors. Only the disease burden associated with high BMI is available for examination in this study. Future research should explore other significant risk factor data, such as injuries and long-term joint load, to enhance the comprehensiveness of findings. Second, the data collected in the GBD database are sourced from diverse countries or regions, which are constrained by variations in medical care levels across different locations. Additionally, a lack of standardized diagnostic and treatment protocols for diseases leads to disparities in data quality that can impact result accuracy. Finally, the SDI is utilized in this study to reflect the socio-economic status of a country or region; however, cultural factors and lifestyles are not thoroughly taken into account.

## Conclusions

In summary, the incidence and prevalence of OA are increasing among younger populations. Previous research has focused on the elderly population, potentially overlooking the disease burden presented by younger people. Therefore, greater emphasis should be placed on developing appropriate intervention measures and proactively implementing effective health management strategies to enhance control over modifiable risk factors, such as optimizing unhealthy lifestyles and maintaining a healthy BMI. This has significant implications for mitigating escalating incidence rates, alleviating disease burden, and enhancing the population’s quality of life.

### Electronic supplementary material

Below is the link to the electronic supplementary material.

Supplementary Material 1

Supplementary Material 2

Supplementary Material 3

Supplementary Material 4

## Data Availability

The data for this study are accessible via a publicly available open-access repository. The datasets utilized for the analyses can be obtained through the GBD Data Input Sources Tool (https://ghdx.healthdata.org/gbd-2019/data-input-sources) and GBD Result Tool (https://vizhub.healthdata.org/gbd-results/).
